# The Influence of Martensitic Intercalations in Magnetic Shape Memory NiCoMnAl Multilayered Films

**DOI:** 10.3390/e23040462

**Published:** 2021-04-14

**Authors:** Andreas Becker, Daniela Ramermann, Inga Ennen, Björn Büker, Tristan Matalla-Wagner, Martin Gottschalk, Andreas Hütten

**Affiliations:** Faculty of Physics, Bielefeld University, P.O. Box 100131, 33501 Bielefeld, Germany; abecker@uni-bielfeld.de (A.B.); dramermann@uni-bielefeld.de (D.R.); ennen@physik.uni-bielefeld.de (I.E.); bbueker@physik.uni-bielefeld.de (B.B.); tristan@physik.uni-bielefeld.de (T.M.-W.); martin.gottschalk2211@gmail.com (M.G.)

**Keywords:** magnetic shape memory alloys, NiCoMnAl alloys, magnetocaloric multilayered films, martensitic intercalations

## Abstract

Hysteresis and transformation behavior were studied in epitaxial NiCoMnAl magnetic shape memory alloy thin films with varying number martensitic intercalations (MIs) placed in between. MIs consists of a different NiCoMnAl composition with a martensitic transformation occurring at much higher temperature than the host composition. With increasing number of intercalations, we find a decrease in hysteresis width from 17 K to 10 K. For a large difference in the layers thicknesses this is accompanied by a larger amount of residual austenite. If the thicknesses become comparable, strain coupling between them dominates the transformation process, which manifests in a shift of the hysteresis to higher temperatures, splitting of the hysteresis in sub hysteresis and a decrease in residual austenite to almost 0%. A long-range ordering of martensite and austenite regions in the shape of a 3D checker board pattern is formed at almost equal thicknesses.

## 1. Introduction

Mechanical stress, temperature, hydrostatic pressure and external magnetic fields are able to induce the shape memory effect in magnetic shape memory alloys [1], making them potentially suitable for many different applications. Thus, their applicability in the fields of microfluidic pumps [2], actuators [3] and artificial multiferroics [4] have been studied extensively in the last years. In particular, endeavors have been made to incorporate them into magnetocaloric refrigeration [5], as such systems have the potential to outperform conventional refrigeration. The demand for such energy efficient systems is constantly rising. In spite of these efforts, many challenges still remain in these materials such as their brittleness [6] and a large thermal hysteresis accompanying the lattice transformation [7]. The latter is the limiting factor for energy efficiency in magnetocaloric cooling systems to be realized with magnetic shape memory materials. Therefore, solutions to this problem need to be developed.

The martensitic transformation (MT) is the origin of the shape memory effect in these materials. It is a reversible, first-order, diffusion less phase transition from the high temperature austenite state to a low temperature phase with lower crystal symmetry called martensite. Especially the large thermal hysteresis during this process is often a limiting factor for applications and needs to be as small as possible. A large undercooling is often necessary, since the martensite state has first to nucleate in the austenite matrix creating phase boundaries with high elastic energy. Upon further cooling the nuclei grow by relocating their phase boundaries along their surface normal, which requires only minimal additional energy [8].

Existing studies try to solve this issue either by optimizing the chemical composition of the material system of interest [9], applying hydrostatic pressure to enhance the phase compatibility between martensite and austenite [10] or just cycling through minor loops of the thermal hysteresis [8,11]. The latter method is based on the idea that the reverse MT is stopped before the full austenite state is reached. Thus, the nucleation step is avoided as martensite nuclei are always present in the minor loop cycle. A physically very similar concept followed in this work is the use of martensitic interlayers alternating with martensitic layers in thin film stacks. This creates a system of two materials, an active transformation layer (AL) with an MT below a working temperature and a martensitic intercalation (MI) with a transition temperature well above the selected working temperature. Just as in the minor loop concept, martensite is always present, which serves as a nucleus when passing through the thermal hysteresis of the AL. Overall, this should reduce the thermal hysteresis of the entire layer stack.

One family of alloys that show the magnetic shape memory effect and often have a broad martensitic transition are the NiMn-based Heusler compounds (Ni_2_MnX, X = {Al, Ga, In, Sn, Sb}) [12,13,14,15]. In off-stoichiometric Ni_50−*x*_Co*_x_*Mn_25+*y*_Al_25−*y*_*,* which undergoes a cubic-to-tetragonal transition, the MT temperature can be shifted by only slightly varying the Al or Co content [15,16]. This offers the advantage that the entire intercalation system can be built with the same material system. In addition, the geometrically very controlled distribution of the martensite cores due to the layer stacking could be advantageous for the transformation behavior. Moreover, in contrast to the minor loop concept, the entire transformation cycle of the AL becomes usable. For our intercalation systems, the working temperature range is thus defined solely by the choice of composition. For the class of MnNiSi-based alloys, for example, it has already been shown very successfully [17] that an adaptation of the working temperature ranges and the associated magnetocaloric effects can also be achieved by heat treatment or the application of hydrostatic pressure.

Little is known yet about the influence on the transformation behavior of martensitic intercalations. Therefore, we investigate these effects in sputter deposited epitaxial thin film multilayer systems and characterize the MT in terms of hysteresis width, transformation temperature and residual austenite. Since in this work we focus strictly on the description of the microstructure-property relationship of the investigated intercalation material systems, the more application-relevant aspects concerning the temperature thermal management of these magnetocaloric materials will not be mentioned further. We would therefore like to refer to a very comprehensive work [18] in which all these aspects are discussed both from their physical origin and in terms of magnetocaloric application near room temperature.

## 2. Preparation of the Multilayered Thin Films and Experimental Methods Utilized

Thin film NiCoMnAl multilayer systems were prepared in a magnetron co-sputter deposition chamber from 3” pure elemental Ni (DC), Mn (DC). Al (DC) and Co (RF) targets with a base pressure better than 5 × 10^−9^ mbar and a target to substrate distance of 21 mm. MgO (001) was used as the substrate material and was heated up in the deposition chamber to 450 °C prior to the deposition of the thin films. In previous studies we found that a less rigid seed layer than MgO improved the martensitic transformation in thin films [19] and therefore a 30 nm V seed layer was deposited before any of the Heusler films. In order to minimize diffusion effects between the active layers and martensitic intercalations the sample was immediately cooled down to room temperature after the deposition of the Heusler layer. Furthermore, all samples were capped with a 2 nm thick Ru layer to prevent oxidation.

**Figure 1:**
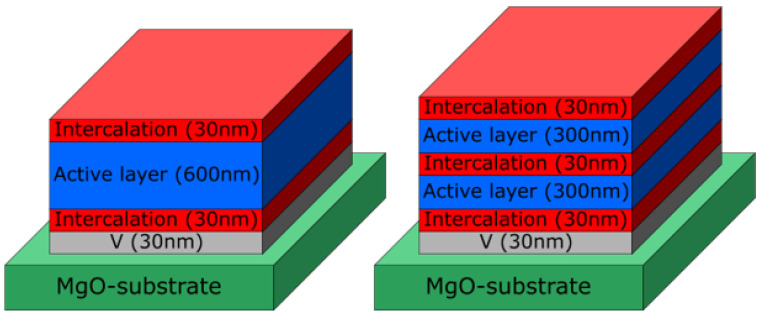
Illustration of the 2 martensitic intercalation (MI) and 3 MI sample systems. The total thickness of the active austenitic layer (600 nm) is divided into equally thick parts, separated by martensitic intercalations with a thickness of 30 nm as shown. All sample systems investigated are capped with 2 nm Ru to prevent oxidation, which is not shown here.

Sample systems were prepared with 0, 2, 3, 4, 7 and 13 MIs. Every intercalation layer had a thickness of 30 nm. The total thickness of the active layers was always held constant at 600 nm and divided into equally thick parts in order to have a constant amount of AL volume throughout the sample series. An illustration of the 2 MI and 3 MI sample systems are shown in Figure 1.

Structural investigations were carried out in a Philips X’pert Pro MPD x-ray diffractometer in Bragg-Brentano geometry using Cu K*α* radiation (XRD). A custom build LN_2_ cryostat with a temperature range from 140 K to 470 K was utilized in order to investigate the crystallography in dependence of temperature. The austenite fraction at a specific temperature was determined by fitting the (004) austenite peak of the Heusler compound with a Pseudo-Voigt function and measuring the area under the curve. The data were then normalized to the measured intensity in the fully austenitic state.

Temperature dependent magnetization measurements in the range from 100 K to 320 K were performed in a 7 T vibrating sample magnetometer (VSM) with an in-plane applied external magnetic field of 500 mT.

High resolution images and selected area electron diffraction (SAED) were recorded in a JEOL FS-2200 transmission electron microscope (TEM). For the TEM-analysis a cross section is cut out from the sample along the [110] MgO direction by means of Ga^+^-ion beam milling at 30 keV and polishing at 5 keV in a FEI Helios DualBeam FIB. The local microstructure of the samples was determined by fast-Fourier transformation (FFT) analysis of the high-resolution images. Furthermore, diffusion effects were investigated by energy dispersive x-ray spectroscopy (EDX) mapping of the samples cross section.

## 3. Results and Discussion

### 3.1. Choosing the Austenitic and Martensitic Composition of the Individual Layers of the Multilayer Stack

**Figure 2:**
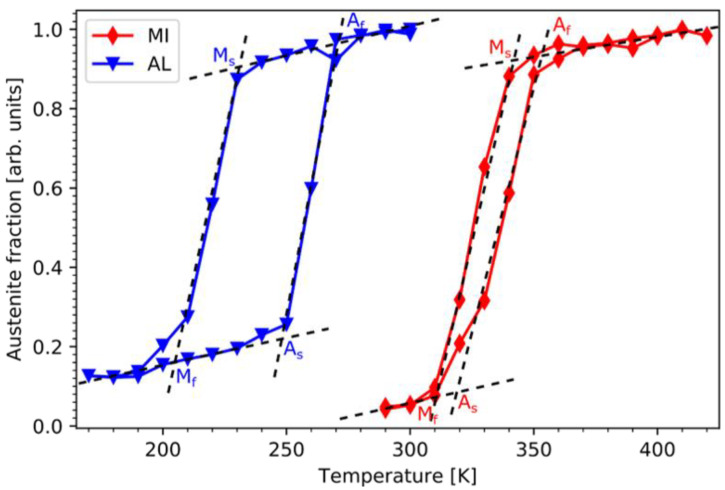
As desired, the thermal hystereses for the active austenitic Ni_43_Co_7_Mn_31_Al_19_ layers (blue) and for the martensitic Ni_47_Co_3_Mn_33_Al_17_ interlayers (red) show no overlap. Thus, these compositions form the starting point for the realization of the multilayer stacks with increasing number of layers. The values for the corresponding transformation temperatures, as given here, are summarized in Table 1.

For convenience room temperature (RT) was chosen as reference working point for this study. Implementing the idea of producing multilayer stacks with alternating austenitic and martensitic layers then requires a composition with a MT below and a composition above RT. For this purpose, 200 nm thick NiCoMnAl films with different compositions were deposited and their transition temperatures were determined by temperature-dependent XRD measurements. Nominal compositions Ni_43_Co_7_Mn_31_Al_19_ for the active austenitic layer and Ni_47_Co_3_Mn_33_Al_17_ for the martensitic intercalations had been identified. Their respective thermal hysteresis is shown in Figure 2. The AL (blue triangles) shows a very wide hysteresis between 200–250 K, while the MI (red diamonds) is characterized by a very small hysteresis between 320–340 K. Both underlying lattice transformations are completed at RT and thus show no overlap of their martensitic transitions. The values for the corresponding transformation temperatures, as given in Figure 2, are summarized in Table 1.

**Figure 3:**
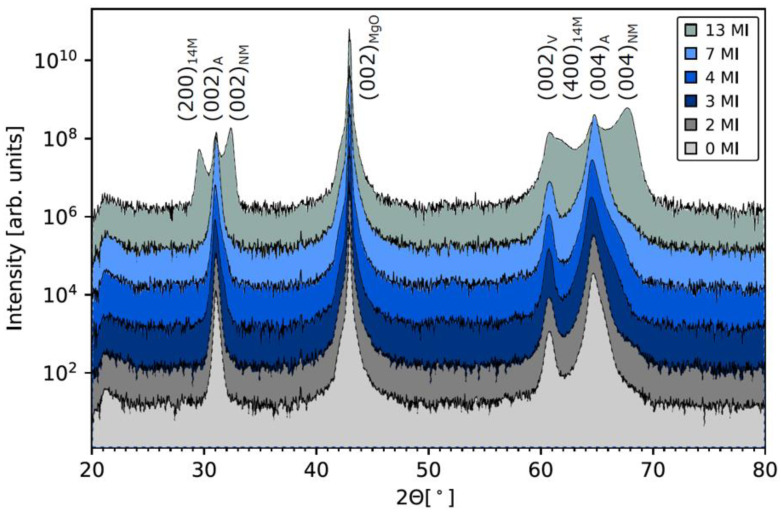
XRD-θ/2θ-measurements at room temperature for the series of all intercalation’s samples.

Figure 3 summarizes the Cu Kα radiation (XRD)-θ/2θ-scans of all the investigated samples to determine their structural properties.

In all scans clearly visible are the (002) MgO peak at 42.91°, the (002) V peak at 60.73° as well as the diffraction peaks (002)_A_ at 30.97° and (004)_A_ at 64.54° associated with the austenite phase. The resulting austenite lattice can be described as a cubic B2-structure with a lattice constant of *a* = 5.771 Å. Martensite features from the intercalations are only present in the 13 MI sample. At 32.36° the (200)_NM_ and at 67.68° the (400)_NM_ peak are visible from the nonmodulated martensite unit cell. Furthermore, to the left of both austenite peaks there is also a diffraction peak visible which could be attributed to the 14M modulation of the martensite, which frequently occurs in this alloy to reduce the interfacial energy between the martensite and austenite [14,20]. The lattice parameters were found to be *a* = 5.53 Å and *c* = 6.62 Å. The notation is given in the form of a tetragonal distorted L2_1_-notation.

### 3.2. Temperature Profile of the Martensitic Transformation in All Multilayer Stacks Measured Using the Temperature Dependence of the Underlying Magnetic Moment

**Figure 4:**
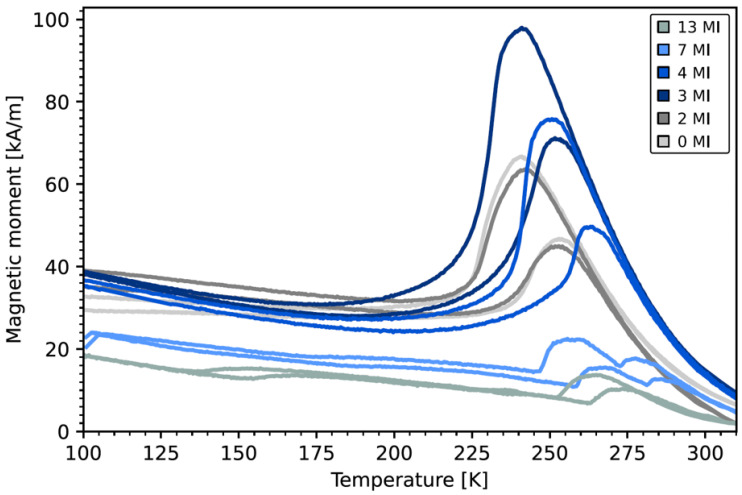
The temperature profile of the magnetic moment at an external magnetic field of 500 mT applied in the sample plane.

**Figure 5:**
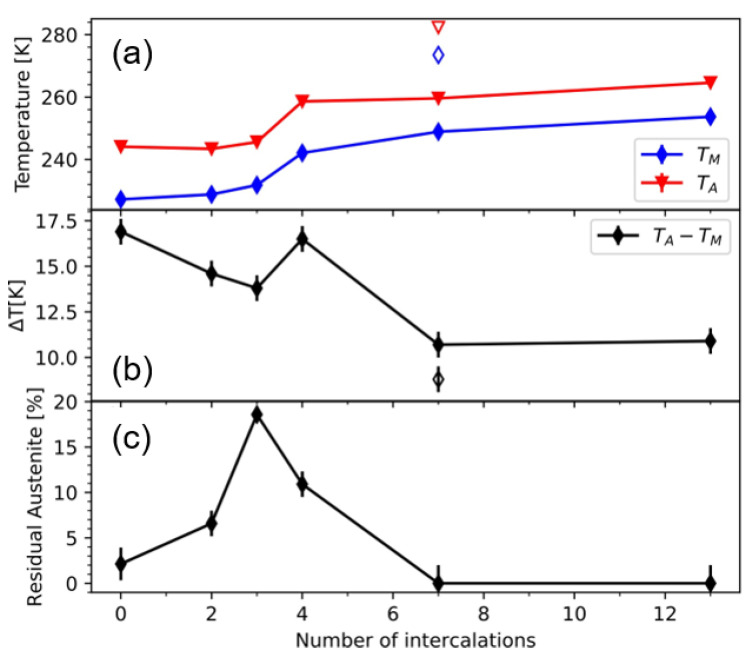
(**a**) Transition temperatures, (**b**) hysteresis width and (**c**) residual austenite content in dependence of the number of martensitic intercalations. The open symbols are the values for the second hysteresis in the 7 MI sample.

Characterization of the martensitic transformation was performed by temperature dependent magnetization measurements in the range from 100 K–320 K with an external in-plane magnetic field of 500 mT and which are given in Figure 4. We find a much different transformation behavior for the 4, 7 and 13 MI samples compared to the 0, 2 and 3 MI sample systems. Starting with the first three samples of the series we find a sharp martensitic transformation occurring around 230 K. The resulting values for the martensitic transformation temperature TM and reverse transformation temperature TA, which are defined by the turning points of the cooling and heating branch respectively, as well as the hysteresis width defined by ∆T = T_A_ − T_M_ are shown in Figure 5a,b. With increasing number of intercalations, we find a steady decrease in the hysteresis width from 16.9 K to 13.8 K. Looking at the transformation temperatures we find that T_M_ remains constant at 278 K while T_A_ shifts to higher temperatures. In this regard the martensitic intercalations act in a similar fashion as the minor-loops in Ni_2_MnGa thin films [8] do.

**Figure 6:**
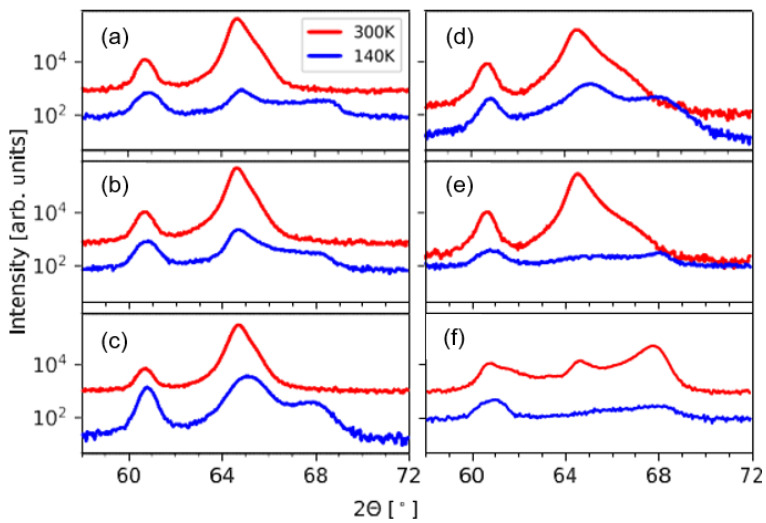
XRD-θ/2θ-scans in the range 58° to 72° at 300 K (red graph) and 140 K (blue graph) for the (**a**) 0 MI, (**b**) 2 MI, (**c**) 3 MI, (**d**) 4 MI, (**e**) 7 MI and (**f**) 14 MI samples.

Another important quantity, which characterizes the martensitic transformation is the amount of residual austenite at low temperatures is quantified and given in Figure 5c. The data points are calculated from XRD measurements of the (004)_A_ peak at 140 K and normalized to measurements at room temperature given in Figure 6. In contrast to the decrease in hysteresis width we find in this case a steady increase in residual austenite from 2.5% to almost 20% for the 3 MI sample. In thin film systems it was shown that the martensite nuclei take the shape of flat, elongated diamonds with their phase boundary inclined by a few degrees from the {011}_A_ directions, which mainly grow in length not in width during martensitic transformation [21]. The nuclei stop to grow if the tip of the diamonds touch other phases, such as substrates or other martensite nuclei, as often an energetically unfavorable incoherent interface has to be formed. Thus, in thin films the thickness of the film defines the maximum size of a martensitic nucleus [22]. As the thicknesses of the ALs become smaller with increasing number of intercalations the maximum size of the martensite nuclei is reduced as well, leaving a lot of untransformed material in between the nuclei. In this regard the MIs can be viewed as barriers for the growth of the martensite. It is unclear at the moment if the martensite nuclei in the MI layers simply grow during cooling and protruding inside the AL layers or if different martensitic nuclei begin to grow inside the active layers, which is facilitated by local strain fields of the MI layers.

The situation drastically changes for the 4, 7 and 13 MI sample systems in which the thicknesses of the ALs approaches the thickness of the MIs. Notably is the sudden shift in the martensitic transformation closer to room temperature. Furthermore, in the 7 and 13 MI samples a splitting of the hysteresis in multiple ones is observed, indicating the existence of regions with different transformation behavior. Still, except for the 4 MI sample, the hysteresis width reduces further to 10.7 K.

The residual austenite content at low temperatures decreases and remarkably for the 7 and 13 MI samples no intensity from the (004)A peak could be distinguished from the background. Thus, in these two samples the amount of residual austenite can be neglected. Since the composition of the two components of the intercalation systems does not change over the sputtering conditions, the increase of the transition temperature of the total layer systems is due to the elastic interaction of these components. What remains to be ensured for this relationship is that the chemical integrity of the components is also not disturbed by interdiffusion of the elements. Furthermore, in our experiments we applied a constant external in-plane magnetic field of 500 mT and worked with a constant heating rate. Thus, in this study we cannot make any statements about the possible influence of magnetic field or heating rate changes on resulting kinetic effects [23] during phase transformation. The investigation of these influences’ merits further study. A fundamental question arises whether an increase in ΔS, which is physically equivalent to a shift in the martensitic transformation closer to the Curie temperature [23], always leads to a reduction in the hysteresis width. This fundamental question can only be answered conclusively when an energy consideration of all involved martensitic defect structures is known. This effort may be possible by detailed DFT simulations.

### 3.3. The Issue of Interdiffusion across the Interfaces of the Multilayer Stacks

**Figure 7:**
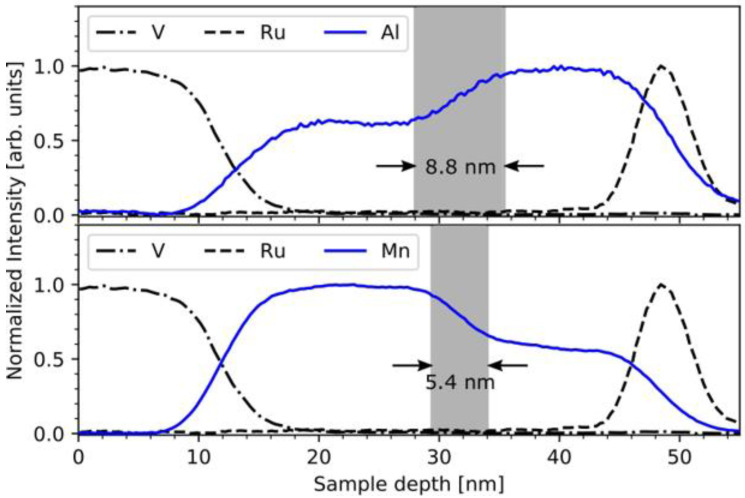
Normalized concentration line profile of the test sample for Al (**upper panel**) and Mn (**lower panel**) calculated from the X-ray spectroscopy (EDX)-mapping.

Since the deposition of the samples takes place at elevated temperatures, diffusion has to be taken into account. Especially the light elements Al and Mn tend to diffuse easily. In order to estimate this effect a 30 nm thick sample was deposited with the same composition of the ALs at the same deposition temperature. To get a high difference in chemical composition the power at Mn source as instantly halved and at the same time the power of the Al source doubled after half of the deposition time. The sample stays heated the same amount of time as the 13 MI sample. Thus, the sample should be representative for the bottom most AL/MI-interface in the 13 MI sample as well as serve as a worst-case scenario for all other interfaces. A line profile is calculated from EDX-mapping of a cross section. Diffusion between the ALs and MIs can be ruled out as the reason for the shift in transformation temperatures. From the normalized line profiles shown in Figure 7 we find that only a region of about 9 nm for Al and 5.5 nm for Mn around the interface is affected in the worst case. Thus, most of the inner part of all layers should be intact.

### 3.4. The Structural Integrity of the Multilayer System Is Lost to Form a 3D Chessboard-Like Microstructure for in the 13 MI Sample

**Figure 8:**
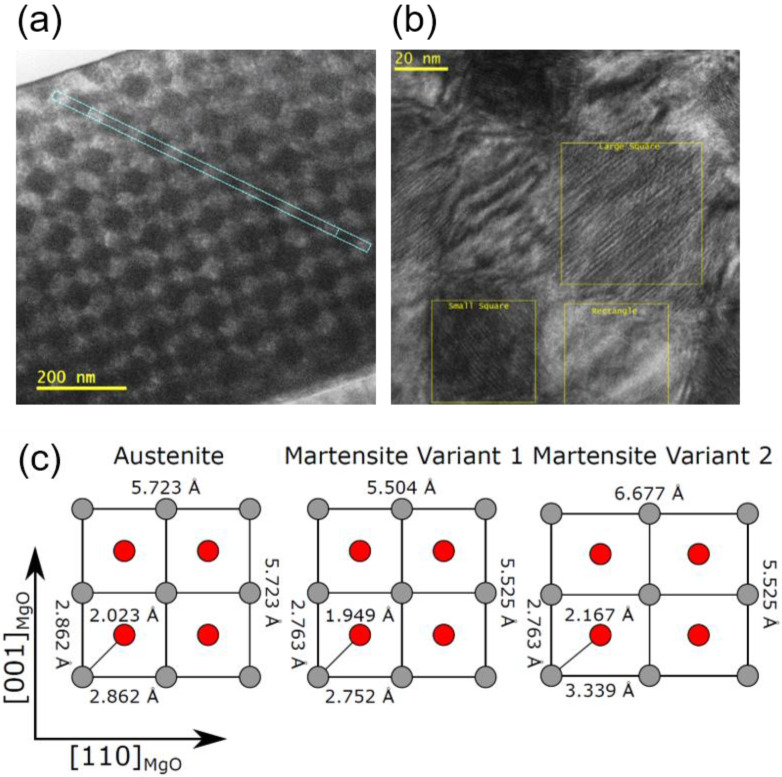
Transmission electron microscope (TEM) images of a cross section from the 13 MI sample. (**a**) The phase contrast of three different regions arises from different crystal structures, some of which overlap, resulting in complex Moiré patterns. The geometric dimensions of these areas have been measured along the white line profiles. (**b**) Details of the high-resolution chessboard, a brightly imaged rectangular area without Moiré contrast, a large dark square area and a small even darker square area, both marked by Moiré patterns. (**c**) Possible austenitic and martensitic crystal structures forming the atomic lattice of the different areas of the 3D chessboard: The austenite lattice forms the lattice structure of the rectangular domains, the superposition of the austenite with the martensitic variant 2 forms the lattice of the large square domains and finally a superposition of the austenite with both martensitic variants forms the lattice of the small very dark domains.

As the number of intercalated layers increases, the integrity of the multilayer system is lost to form a 3D chessboard-like microstructure with 13 intercalation layers. This novel microstructure can be beautifully imaged in high-resolution transmission electron microscopy and is shown in Figure 8. Instead of the intended layered structure a periodic arrangement of darker and lighter regions is observed in cross sections along the [100] MgO and [110] MgO direction, resulting from the elastic coupling between the different layers rearranging the martensite nuclei and controlling their growth during cooling. This leads to a very interesting long range ordering phenomenon as can be seen from cross sections shown in Figure 8a,b. The chessboard consists of three different areas as indicated in Figure 8b. Visible are two squared regions of different sizes and brightness separated by rectangles. Both square regions are oriented that their edges are inclined at (45 ± 2)° to the sample surface, as shown in Figure 8a. Moreover, parallel Moiré patterns are observed in these two regions, but not in the rectangular ones. It is noteworthy that the Moiré patterns of the square regions are perpendicular to each other and thus parallel or perpendicular to the sample surface. By means of a line profile over several cells of the chessboard, compare with Figure 8a, the geometric dimensions of the identified regions have been determined. The edge length of the large square regions is (50 ± 5) nm while for the small squares an edge length of (44 ± 4) nm has been measured. The edges of the rectangular regions are (50 ± 5) nm × (44 ± 4) nm. From the selected area electron diffraction of these three regions and their FFT analysis, the underlying and partially overlapping crystal lattices of the phase and Moiré contrast could be determined.

In detail, this analysis goes far beyond the scope of this paper and will therefore be published in a more microscopically relevant context. However, the result of such an analysis is summarized in Figure 8c. A purely austenite lattice forms the lattice structure of the rectangular domains, the superposition of the austenite with the martensitic variant 2 forms the lattice of the large square domains and finally a superposition of the austenite with both martensitic variants forms the lattice of the small very dark domains. Thus, the analysis confirms that the contrast between the checkered areas is caused by the presence of different martensite and austenite unit cells. The rectangular areas, which show no Moiré patterns, are composed of austenite only. In contrast, the two square areas each have martensite nuclei with different martensite orientations that are masked by the austenite in front and behind them, respectively, which is possible due to the thickness of the TEM lamella. However, the exact 3D shape of these nuclei is still unclear at the moment.

## 4. Summary and Conclusions

We prepared multilayer systems consisting of two different NiCoMnAl compositions with one having a MT above and one having a MT below room temperature. The transformation behavior of the layers with MT below RT were investigated. We find that the hysteresis width almost steadily decreases if more intercalations are present, thus these can facilitate the growth of martensite in the ALs. Two different transformation ranges can be distinguished. If the ALs are significantly thicker than the MIs the transformation temperature for the martensitic transformation shifts to higher temperature while the reverse transformation is unaffected. However, the intercalations might also serve as barriers, which limit the maximum size of the martensite nuclei in the ALs. Thus, with increasing numbers of intercalations more residual austenite remains present at low temperatures. If the thicknesses become comparable elastic coupling between the layers dominates the transformation process, which results in an ordered distribution of martensite and austenite regions.

## Figures and Tables

**Table 1:** Martensite start M_s_, martensite finish M_f_, austenite start A_s_ and austenite finish A_f_ temperatures for the active transformation layer (AL) and MI thermal hysteresis shown in Figure 2.

Layer	M_s_ [K]	M_f_ [K]	A_s_ [K]	A_f_ [K]
AL	232	204	248	271
MI	342	310	319	353

## Data Availability

Not applicable.
